# Food allergy in small children carries a risk of essential fatty acid deficiency, as detected by elevated serum mead acid proportion of total fatty acids

**DOI:** 10.1186/1476-511X-13-180

**Published:** 2014-12-02

**Authors:** Marita Paassilta, Elina Kuusela, Matti Korppi, Riina Lemponen, Minna Kaila, Seppo T Nikkari

**Affiliations:** Allergy Centre, Tampere University Hospital, Tampere, Finland; Paediatric Research Centre, Tampere University and University Hospital, Tampere, Finland; School of Health Sciences, University of Tampere, Tampere, Finland; Hjelt Institute, University of Helsinki, Helsinki, Finland; University of Tampere School of Medicine and Fimlab Laboratories, Tampere, Finland

**Keywords:** Essential fatty acid deficiency, Food allergy, Child, Elimination diet, Mead acid

## Abstract

**Background:**

Elevated serum Mead acid as a proportion of total fatty acids is an indirect marker of a deficiency of essential fatty acids (EFA). The aim of the study was to evaluate the symptoms and nutrition of food-allergic children with elevated or normal serum Mead acid.

**Methods:**

Serum fatty acid compositions from 400 children were studied by clinical indications, mostly by suspicion of deficiency of EFA due to inadequate nutrition. A Mead acid level exceeding 0.21% (percentage of total fatty acids) was considered to be a specific sign of an insufficient EFA supply. From a total of 31 children with elevated Mead acid (MEADplus group), 23 (74%) had food allergy. The symptoms and dietary restrictions of this MEADplus group of food allergic children were compared to 54 age-and sex-matched controls with food allergy but normal Mead acid proportions (MEADminus group) before and 6 months after the serum fatty acid determination.

**Results:**

At the beginning of the 6-month follow-up, 44% of the food allergic children in both MEADplus and MEADminus groups were on an elimination diet. These diets did not differ between the two groups and we were not able to document an association between the severity of elimination diet and elevated Mead acid proportion. However, the MEADplus children were on average more symptomatic than MEADminus children. In the MEADplus group, food allergy presented with skin symptoms in 100% (vs. 70% in the MEADminus group, p < 0.001) and with vomiting or diarrhea in 70% (vs. 44% in the MEADminus group, p < 0.05). Clinical suspicion of malnutrition resulted in increase in the use of vegetable oil and milk-free margarine in both groups from <50% to 65-74% during the follow-up. After 6 months, 64% of the MEADplus children with food allergy had been sent to a control serum fatty acid analysis. Of these children, Mead acid had declined to normal level in 69%, and remained elevated in 31%.

**Conclusions:**

Severe symptoms of food allergy combined with elimination diets in children may lead to insufficient nutrition presenting with elevated serum Mead acid. Adding of supplementary polyunsaturated fat to the diet should be considered in these children.

## Background

Elimination diets form the basis of the management of clinically significant food allergy. Dietary limitations carry the risk of insufficient nutrition, especially when foods important for children, such as milk, egg, fish, grain and vegetables, are eliminated, as documented *e.g*. by impaired growth and failure to thrive [1]. Food allergic children should be monitored for growth, and guiding is essential for choosing appropriate alternatives to supply necessary nutrients [2].

Essential fatty acids (EFA) are fatty acids that the human body is not able to synthesize, and therefore, they must be obtained from the diet. Fat supply especially that of EFAs, may be insufficient in children with restricted diet due to different malabsorption syndromes and even due to food allergy [3–5]. If the EFA intake from the diet is not sufficient, the liver produces long-chain polyunsaturated fatty acids from oleic acid, and the end product is 5,8,11-eicosatrienoic acid (Mead acid) [6]. In an experimental study on cultured cells, an induced state of essential fatty acid depletion resulted in a pronounced emergence of Mead acid [7]. Thus, the production of Mead acid is indirectly associated with the supply of the EFAs in the diet, and an elevated Mead acid in serum is an indicator of essential fatty acid deficiency [4, 8, 9]. The genes and pathways involved in the synthesis of Mead acid (20:3n-9) were identified just recently [10].

The aim of the present paper was to evaluate the association between symptoms, dietary restrictions and serum Mead acid concentration in children with food allergy.

## Results

**Figure 1:**
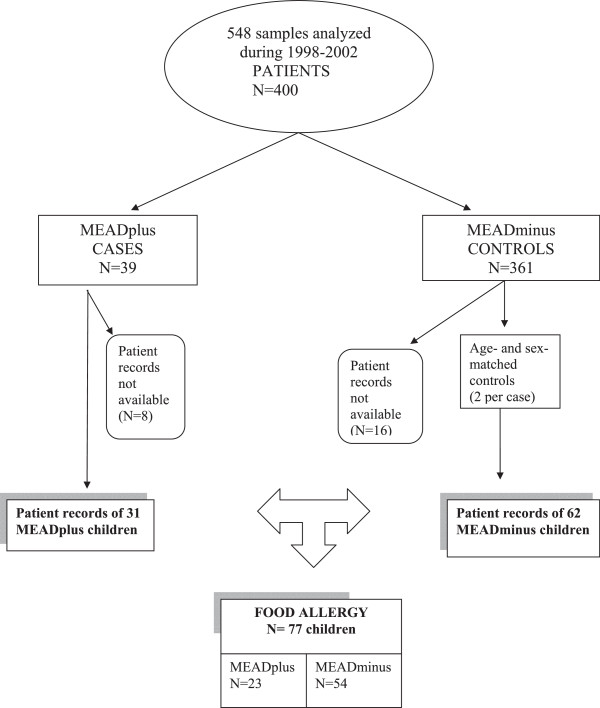
**Design of the study.**

Of the studied 400 children who had been sent to serum fatty acid analysis on clinical bases, 39 had a Mead acid level exceeding 0.21% (percentage of total fatty acids), which was considered to be a specific sign of an insufficient EFA supply [8] (Figure 1). Patient records were available for 31 of them, and subsequently 23 children (74%) were found to have food allergy. They formed the MEADplus group. The MEADminus group was formed from 54 food allergic children with Mead acid in the normal range.

There were 20 boys (87%) and 3 girls in the MEADplus group. The median age of the MEADplus children was 1.5 years (range 0.6 – 4.6). In the MEADminus group of 54 children, there were were 39 boys (72%) and the median age was 1.4 years (0.5 – 4.6). In the MEADplus group, food allergy presented with skin manifestations in 100% (vs. 70% in the MEADminus group, p < 0.001), with vomiting or diarrhea in 70% (vs. 44%, p < 0.05) and with respiratory manifestations in 44% (vs. 54%, p = NS) (Table 1). However, cow`s milk challenge, skin prick tests to cow`s milk and specific IgE to cow`s milk were positive more often in the MEADminus than in the MEADplus group (Table 2).

**Table 1:** **Manifestation of food**-**allergy symptoms in the groups of 23 MEADplus and 54 MEADminus children**

	MEADplus ^1^ n (%)	MEADminus ^2^ n (%)	Statistical significance
Skin - eczema	23 (100)	38 (70)	p < 0.001
Gastrointestinal	16 (70)	24 (44)	p < 0.05
Respiratory	10 (44)	29 (54)	p = NS

^1^Mead acid ≥0.22% of serum fatty acids; ^2^Mead acid <0.22% of serum fatty acids.

**Table 2:** **Positive allergy test results of the food-allergic children in the MEADplus and MEADminus groups**

	MEADplus ^1^ (n = 23) n (%)	MEADminus ^2^ (n = 54) n (%)	Statistical significance
Milk challenge	11/17 (65)	44/47 (94)	p = 0.008
Wheat challenge	6/13 (46)	20/37 (54)	p = 0.624
Milk skin prick test	4/18 (22)	23/47 (49)	p = 0.050
Wheat skin prick test	8/22 (36)	24/49 (49)	p = 0.323
Milk-specific IgE	6/22 (27)	31/46 (67)	p = 0.002
Wheat-specific IgE	8/21 (38)	27/49 (55)	p = 0.192

^1^Mead acid ≥0.22% of serum fatty acids; ^2^Mead acid <0.22% of serum fatty acids.

In the MEADplus group, 5/23(22%) children consumed 5-10 foodstuffs (vs. 7/54(13%) in the MEADminus group), and 5/23(22%) consumed <5 foodstuffs (vs. 17/54(31%) in the MEADminus group), with no statistically significant differences between the groups. The detailed diets are presented in Table 3, again with no significant differences between MEADplus and MEADminus groups. Six months later, at the median age of 2.0 years (range 1.06 – 5.13), 4/23(17%) children in the MEADplus group still consumed 5-10 foodstuffs but none consumed <5 foodstuffs. In the MEADminus group, the respective figures were 6/54(11%) and 2/54(4%) at the median age of 2.0 years (range 0.83 – 5.26), with no significant differences between MEADplus and MEADminus groups. The most often avoided foodstuffs included milk, cereals, egg and fish. As seen in Table 3, the use of vegetable oil and milk-free margarine had increased from <50% to 65-74%. At the end of follow-up, as many as 7(30%) used no milk product in the MEADplus group. In the MEADminus group 5 (9%) used no milk product.

**Table 3:** **Diets of the MEADplus and MEADminus food allergic children prior to and 6 months after the fatty acid (FA) analysis**

	Diet prior to FA analysis		Diet 6 months after FA analysis	
	MEADplus ^1^ (n = 23) n (%)	MEADminus ^2^ (n = 54) n (%)	MEADplus ^1^ (n = 54) n (%)	MEADminus ^2^ (n = 54) n (%)
Elimination diet	10 (44)	24 (44)	4 (17)	8 (15)
<5 foodstuffs	5 (22)	17 (31)	0 (0)	2 (4)
5-10 foodstuffs	5 (22)	7 (13)	4 (17)	6 (11)
Fish	1 (4)	0 (0)	2 (9)	5 (9)
Vegetable oil	11 (48)	25 (46)	16 (70)	37 (74)
Milk-free margarine	10 (43)	25 (46)	15 (65)	40 (74)
Formula only	1 (4)*	2 (9)**	0 (0)	0 (0)

^1^Mead acid ≥0.22% of serum fatty acids; ^2^Mead acid <0.22% of serum fatty acids. *One patient on hydrolyzed casein formula only. **One patient on amino-acid formula only. There were no statistically significant differences between the MEADplus and MEADminus groups at either prior to FA analysis or after FA analysis.

After 6 months, 13 (64%) of the 23 MEADplus children with food allergy had been sent to a control serum fatty acid analysis. Of these children, Mead acid had declined to normal level in 9 (69%), and remained elevated in 4 (31%). In total, only 9 (39%) of the 23 MEADplus children with food allergy were documented to have normal Mead acid after 6-month follow-up.

## Discussion

The results of the present laboratory-based study suggest that there is a real risk for insufficient supply of EFAs in young children with food allergy. This insufficient supply was indicated indirectly, by measuring serum Mead acid proportions in 400 children aged on average under two years, who were considered to be at risk for insufficient nutrition. Serum levels of Mead acid, the hallmark acid of EFA deficiency, were elevated in 10% of the children, and among them 74% had food allergy. The other reasons were severe malabsorption syndromes, short bowel syndrome and nutrition therapy of familial lipid disorders, *i.e*. well-known causes of malnutrition and conditions reported to be related to elevated Mead acid appearance [3].

The omega-3 fatty acid, α-linolenic acid (ALA), and omega-6 fatty acid, linoleic acid (LA), are essential for optimal development and health. In the human body, LA (omega-6) is metabolized to long-chain metabolites dihomo-γ-linolenic acid (DGLA) and arachidonic acid (AA). Omega-6 fatty acids are abundant in nuts and seeds but are also present in poultry, eggs, cereal and in whole-grain breads. ALA (omega-3) is converted to eicosapentaenoic acid (EPA) and very poorly to docosahexaenoic acid (DHA) [11]. The most widely available sources of omega-3 fatty acids are cold-water oily fishes, such as salmon, herring and sardines. Other sources are kiwi fruit, lingonberry, nuts, eggs and linseeds, and especially grass-fed animals and milk or cheese of grass-fed cows. In marine food -consuming populations, the mothers have higher breast-milk concentrations of DHA [11]. Fish is often eliminated in food-allergic young children. Although there are no specific recommendations about fish oil and omega-3 fatty acids for children, it is generally considered important to include fish, nuts, and seeds in a child's diet.

Siguel et al. developed a specific method for plasma EFA status by utilizing capillary-column gas-liquid chromatography technique [8]. They concluded in their laboratory-based study in 56 healthy adults and in 10 adults with inflammatory bowel disease, that an elevated Mead acid concentration (over 0.21% of total fatty acids) in plasma is a rather sensitive and very specific marker of the deficiency of EFAs. By using the same cut-off limit, we were not able to document an association between the severity of elimination diet and the presence of elevated Mead acid concentration in young children. The small sample size of the study did not allow any foodstuff-specific subgroup analyses. In all, the most often avoided foodstuffs included milk, cereals, egg and fish. On the other hand, food allergy presented more often with skin manifestations, vomiting or diarrhea in the MEADplus group then in the MEADminus group. Thus, these symptoms might play an important role through avoidance or malabsorption of nutrients. Unexpectedly, a third of the food-allergic children with elevated Mead acid had no milk available in the diet, though they on the average were less than two years old.

Although the use of fish oil, vegetable oil and milk-free margarine (most often containing rapeseed oil), which are the important sources of omega-3 fatty acids, increased during the 6-month follow-up, still not all (<75%) used these oils appropriately. Moreover, less than half of the MEADplus children with food allergy were documented to have normal Mead acid after 6-month follow-up. Thus, the food-allergic children were at risk for omega-3 fatty acid deficiency at a young age of under two years, which is critical for adequate growth and brain development [12, 13].

Nutritional management, an essential component of the therapy of food allergy, should be carefully planned and monitored. Dietary fat should provide a balanced combination of saturated, monounsaturated and polyunsaturated fatty acids. Because fish and fish oils are often restricted, the addition of vegetable oils to supplement unsaturated fatty acids is advised in most guidelines [14, 15]. However, in the beginning of follow-up, supplemental fat was provided for only half of the children with food allergy in the present study.

The current retrospective laboratory-based study offers only a rough estimate about the lack of EFAs in children with food allergy. The evaluation of the prevalence of elevated Mead acids and insufficient intake of EFAs, and the evaluation of their associations with food allergy and foodstuff-specific diet manipulation, need prospective, population-based studies. A firm diagnosis of food allergy is essential, and should be based on elimination-challenge tests. Once given a diagnosis, the continuing need of an elimination diet should be evaluated repeatedly, following a scheduled program, to avoid prolonged unnecessary dietary restrictions.

## Conclusions

Food allergy in children may lead to insufficient supply of EFA through allergic symptoms and food restriction presenting with elevated serum Mead acid. The essential role of long-chain omega-3 fatty acids is especially important for the brain and the retina, and should be ensured. Adding of supplementary polyunsaturated fat should be considered in diets of food allergic small children.

## Methods

Serum fatty acid compositions, including the concentration of Mead acid, were studied mostly by suspicion of deficiency of EFA due to inadequate nutrition in 400 children (548 samples) in the Laboratory of Medical Biochemistry, Tampere University, Tampere, Finland, during a 5-year period from 1998 to 2002 (Figure 1). Among these 400 children, Mead acid was elevated (≥0.22% of total fatty acids) in one or more serum samples of 39 children.

The proportion of Mead acid (5, 8, 11-eicosatrienoic acid, 20:3n-9) was measured by a fatty acid assay in which the serum lipids were first extracted with chloroform methanol, and then hydrolyzed and methylated. The fatty acid methyl esters (between C14:0-C22:6) were separated, and the percentage distribution was analyzed by capillary gas-liquid chromatography. The Mead acid level exceeding 0.21% (percentage from total fatty acids) was regarded as elevated, and was considered to be a specific sign of an insufficient EFA supply [8].

Data on asthma and allergy, food allergy in particular, and on other underlying illnesses were retrospectively collected from the individual patient records at the Tampere University Hospital or at the central hospitals of the Tampere University Hospital District in accordance with ethical standards of the Helsinki Declaration 2000. The study was a retrospective hospital chart review. The blood samples were not taken particularly for fatty acid analysis but were part of routine clinical assessment of the nutrition status by the physician. Since patients were not contacted, the study was performed by the permission of the Chief Physician of the University Hospital. Appropriate data were available for 31 children with elevated Mead acid percentage (MEADplus group). Sixty-two age- and sex-matched controls were randomly selected from 345 subjects with normal Mead acid levels and available patient records (MEADminus group).

Among the 31 children from the MEADplus and the 62 children from the MEADminus group food allergy was present in 23/31(74%) in the MEADplus and in 54/62(87%) in the MEADminus group. Thus, 16 children in the MEADplus group and 8 in the MEADminus group had another illness than food allergy (the figures for the MEADminus group within parentheses): short bowel syndrome 3(0), familial hypercholesterolemia 1(3), severe malnutrition 1(0), biliary atresia 1(2), phytosterolemia 1(2), long-chain 3-hydroxyacyl-coenzyme A dehydrogenase (LCHAD) deficiency 1(0) and Crohn’s disease 0(1). 

The food-allergic 23 children in the MEADplus and 54 children in the MEADminus group formed the subjects of the present study (Figure 1). In addition to the characteristics of food allergy, detailed data on diet restrictions were collected from the patient records. The dietary data were collected covering the sampling time and the on average 6 months’ period after sampling (median 5.8 months, range 0 – 15). The presence of skin, gastrointestinal (vomiting and/or diarrhea) and respiratory allergies were recorded separately, and only those confirmed by a physician were included. Likewise, data on the results of the elimination-challenge tests, the skin prick tests (positive ≥3 mm reaction) and serum allergen-specific immunoglobulin E (IgE) determinations (positive ≥0.35 units/ml) were collected if available.

Pearson`s chi-square test and Fisher`s exact test were used in the statistical analysis of the data, by applying the SPSS for Windows (version 15.0) statistical package.
